# Improved runoff forecasting based on time-varying model averaging method and deep learning

**DOI:** 10.1371/journal.pone.0274004

**Published:** 2022-09-15

**Authors:** Jinlou Ran, Yang Cui, Kai Xiang, Yuchen Song

**Affiliations:** Henan Provincial Communications Planning and Design Institute Co., Ltd, Zhengzhou, P.R. China; Sant Longowal Institute of Engineering and Technology, INDIA

## Abstract

In order to improve the accuracy and stability of runoff prediction. This study proposed a dynamic model averaging method with Time-varying weight (TV-DMA). Using this method, an integrated prediction model framework for runoff prediction was constructed. The framework determines the main variables suitable for runoff prediction through correlation analysis, and uses TV-DMA and deep learning algorithm to construct an integrated prediction model for runoff. The results demonstrate that the current monthly runoff, the runoff of the previous month, the current monthly temperature, the temperature of the previous month and the current monthly rainfall were the variables suitable for runoff prediction. The results of runoff prediction show that the TV-DMA model has the highest prediction accuracy (with 0.97 Nash-efficiency coefficient (NSE)) and low uncertainty. The interval band of uncertainty was 33.3%-65.5% lower than single model. And the prediction performance of the single model and TV-DMA model in flood season is obviously lower than that in non-flood season. In addition, this study indicate that the current monthly runoff, rainfall and temperature are the important factor affecting the runoff prediction, which should be paid special attention in the runoff prediction.

## Introduction

Runoff simulation is an important means to simulate water cycle [1]. Accurate and reliable runoff prediction can help flood control managers to deal with floods reasonably, which is of great significance to watershed and urban flood control [2]. However, due to the nonlinearity and complexity of runoff series [3], accurate and reliable prediction of runoff is a very challenging task. In addition, the frequent extreme climate events make the runoff series show greater volatility [4], which brings great challenges to the runoff prediction. Therefore, advanced models were needed to provide higher accuracy and more stable runoff prediction [4].

In the past few decades, numerical models of physical mechanism [5–7] and data-driven methods [8–10] have been widely used in runoff prediction and simulation [11]. The numerical model of physical mechanism started early and has been widely used in runoff prediction [12–14]. However, the hydrological process of the basin is very complex. Therefore, accurate hydrological process simulation requires a large number of accurate hydrological process data and fine underlying surface data. [15]. In addition, the calculation of runoff and confluence, model construction and parameter setting mainly depend on modeler’s understanding of the actual hydrological process, resulting in certain subjectivity and uncertainty in the modeling process of the physical model [16]. With the development of artificial intelligence and deep learning technology, runoff series prediction by data-driven method has become popular [17, 18]. The data-driven model does not need to consider the physical mechanism. Through the analysis of time series, it can capture the nonlinear relationship between driving factors and runoff, which can avoid the influence of subjective factors on the uncertainty of the model [19]. Time series prediction models such as Back Propagation (BP) [20], Long Short-Term Memory (LSTM) [21] and Gated Recurrent Unit (GRU) [22] have been successfully applied to the study of runoff prediction.

Due to the non-stationarity of runoff series and the uncertainty of model input, structure and parameters [23–25], there is often great uncertainty in predicting by single model [26]. In other words, the model may achieve high prediction accuracy under one event, but may have poor accuracy under another event. However, the multi-model method [27–29] provides the possibility for stable runoff prediction. Among them, model average method is an effective method to deal with model uncertainty and improve model performance and stability [30, 31], mainly including Bayesian Model Average (BMA) [27] and Frequency Model Average (FMA) [28]. Among them, BMA is one of the most commonly and widely used methods to generate reliable model prediction. In recent years, BMA has been widely used in runoff simulation and prediction [11, 32, 33]. Huo et al. [34] used the numerical model of physical mechanism and BMA to simulate the flood process in semi humid areas. The results demonstrate that the BMA method coupled with multiple numerical models can provide more accurate prediction. Neto et al. [35] integrated BMA and five configuration results of SWAT model to synthesize a probability prediction, which improved the runoff prediction in the area lack of data. Bo et al. [36] applied BMA to the post-processing of multi-mode large set hydrological prediction. The results indicate that BMA can improve the performance of the original multi-mode large set runoff prediction and produce a more calibrated and clearer prediction probability density function in the forecast period of 24 to 120 hours. In addition, the FMA has begun to receive extensive attention in Econometrics and statistics over the past decade [37–39]. Hjort and claeskens [37] established a general large sample likelihood tool, which not only accurately describes the limit distribution and risk characteristics of the average estimator of the model, but also explicitly considers the model deviation and greatly reducing the complexity of the model. Hansen [39] introduced Mallow’s criterion into model averaging and proposed a new FMA method based on Mallow model averaging (MMA), which not only proved the progressive optimality of MMA, but also showed that MMA has advantages over other feasible prediction methods. Chen et al. [38] proposed two model averaging schemes based on exogenous regression and autoregressive lag, aiming to obtain more accurate time series estimation and prediction by using a large number of conditional variables in a nonparametric manner.

As described in the above literature, the model averaging method (BMA and FMA) provides a more robust method for runoff prediction. BMA assigns weights to each model through posteriori probability, which was deduced by priori probability. It can not only provide high-precision multi-model comprehensive prediction, but can quantitatively evaluate the uncertainty of model [40]. The choice of different prior probabilities has a great impact on BMA results. However, there is no unified standard of determining prior probabilities, which resulting that the determination of the prior probability was affected by subjective factors [41]. The FMA determines the weight of each model through combination optimization. The optimal weight was determined by data-driven, avoiding the influence of human factors [28]. However, to the best of the authors knowledge, there are hardly research applying the frequency model average method to runoff prediction. In addition, the weight of each model in the above literature was fixed, but in the process of runoff series prediction, the prediction performance of different models at each time node may vary greatly. In other words, one model may produce the best prediction in one period, but its prediction performance in other periods may be lower than other model [28]. Therefore, the fixed weight may make the model have large errors in local prediction.

Based on this, this study attempts to propose a dynamic model averaging method with Time-varying weights (TV-DMA) based on the idea of frequency model averaging. This method corrected the prediction performance of each model at each time by the translation transformation of the prediction error distribution function. On this basis, the optimal weight of each model was derived by using the minimization error criterion to avoid the subjective influence of the model in determining the initial weight. Finally, the weight at each time was dynamically adjusted through the error propagation mechanism, which aims to build a dynamic model averaging method with time-varying weight.

The innovation of this study was that a dynamic model averaging method with time-varying weight was proposed. Using this method, BP, LSTM and GRU models were integrated, and a comprehensive runoff prediction framework based on TV-DMA and data-driven method was proposed. Firstly, the catastrophe characteristics of runoff prediction series were analyzed by Mann-Kendall (M-K) test [42]. On this basis, the sensitivity indexes suitable for runoff prediction were proposed. And the runoff prediction model was constructed by using these indexes and three data-driven methods. Finally, the integrated runoff prediction model was proposed by TV-DMA and data-driven model, which aims to improve the accuracy of runoff prediction.

## Materials and methods

### Study area and data

The Huaihe River Basin was located in eastern China, with a total drainage area of 0.27 million km^2^. The terrain was high in the northwest and low in the southeast. This basin belonged to the warm temperate zone and semi humid monsoon climate zone, with an annual average temperature of 11°C -16°C. The rainfall in this area was unevenly distributed throughout the year, mainly from June to September [43]. And the annual average precipitation was about 920 mm. In this study, the hydrological station in Xixian was selected as the representative station. The monthly runoff, temperature and rainfall data from 1957 to 2016 were collected. The data was divided into three sample data: flood season, non-flood season and every month. The flood season includes the data from June to September. Runoff, temperature and rainfall data are from Xixian station of Huaihe River Basin. Runoff data refers to the monthly total runoff of Xixian station, temperature data refers to the monthly average temperature of Xixian station, and rainfall data refers to the monthly cumulative rainfall of Xixian station. All data were provided by the Hydrological Bureau of Huaihe River Basin and the Meteorological Department of Henan Province, which can be viewed and obtained in S1 Data.

The runoff series has significant non-stationarity, especially in flood season, which fluctuates violently and cause some uncertainty to the time series prediction. Therefore, in order to eliminate the adverse effects caused by singular sample data as much as possible, the maximum and minimum normalization method [44–46] was used to normalize the runoff, rainfall and temperature data.

### Framework of runoff prediction model based onTV-DMA

The proposed model (TV-DMA) was an integrated forecasting framework to improve the accuracy and stability of runoff forecasting. The framework mainly includes four aspects.

The first is the sample data processing process. In order to minimize the impact of sample uncertainty on runoff prediction, the M-K test was used to select sample data for model training and verification, which is also one of the key steps of the runoff prediction model.

The second aspect was the selection of model input variables. Although scholars generally believed that runoff prediction is closely related to runoff, rainfall and temperature [3], there was no unified standard for which factors were applicable to runoff prediction [47]. For example, the runoff at the next moment is obviously closely related to the current runoff, but the runoff at the previous moment may also affect the runoff at the next moment. Similarly, Earlier runoff may also affect the runoff at the next time. Therefore, it is necessary to adopt appropriate methods to evaluate the impact of these factors on the runoff at the next time, and select the input variables suitable for runoff prediction. Based on this, this study calculated the correlation between runoff and runoff, rainfall, temperature and different lag time of these factors to screen the input variables of runoff prediction.

Thirdly, the selection of benchmark models. Although with the rapid development of computer technology, many deep learning and artificial intelligence algorithms were widely and successfully used in the various fields, not the most advanced methods can be suitable for all research. Runoff prediction was a time series problem. Recent research shows that BP [48], LSTM [3] and GRU [22] approach have been successfully applied to runoff prediction and achieved good results. Therefore, this study selects BP, LSTM and GRU as the benchmark models of TV-DMA model.

The last aspect was the establishment of TV-DMA runoff prediction model. TV-DMA was a dynamic model averaging method with time-varying weights, which depends on the results of each benchmark model. Its basic idea was to use the errors of the prediction results of each benchmark model to give dynamic weights, in order to improve the prediction accuracy and performance of single model.

### Benchmark model

BP, LSTM and GRU neural network were selected as the benchmark model of integrated model. BP is a feedforward neural network widely used in data mining, which mainly includes input layer, hidden layer and output layer [49]. Firstly, initialize the parameters of BP neural network, then take the influencing factors related to runoff as input, and take the runoff as output to construct a neural network for runoff prediction. If the output value of the BP neural network contains errors, the error will be corrected layer by layer from the output layer to the input layer through the error back propagation algorithm. By continuously adjusting the connection weights between layers, the output of the BP neural network is closer to the expected output. The advantage of BP neural network is that it can deal with the mapping relationship between rainfall and runoff. And it has always been a popular choice for handling various complex physical processes in the field of hydrology [48]. The complete mathematical description of BP neural network can be found in [41] and [50].

**Fig 1:**
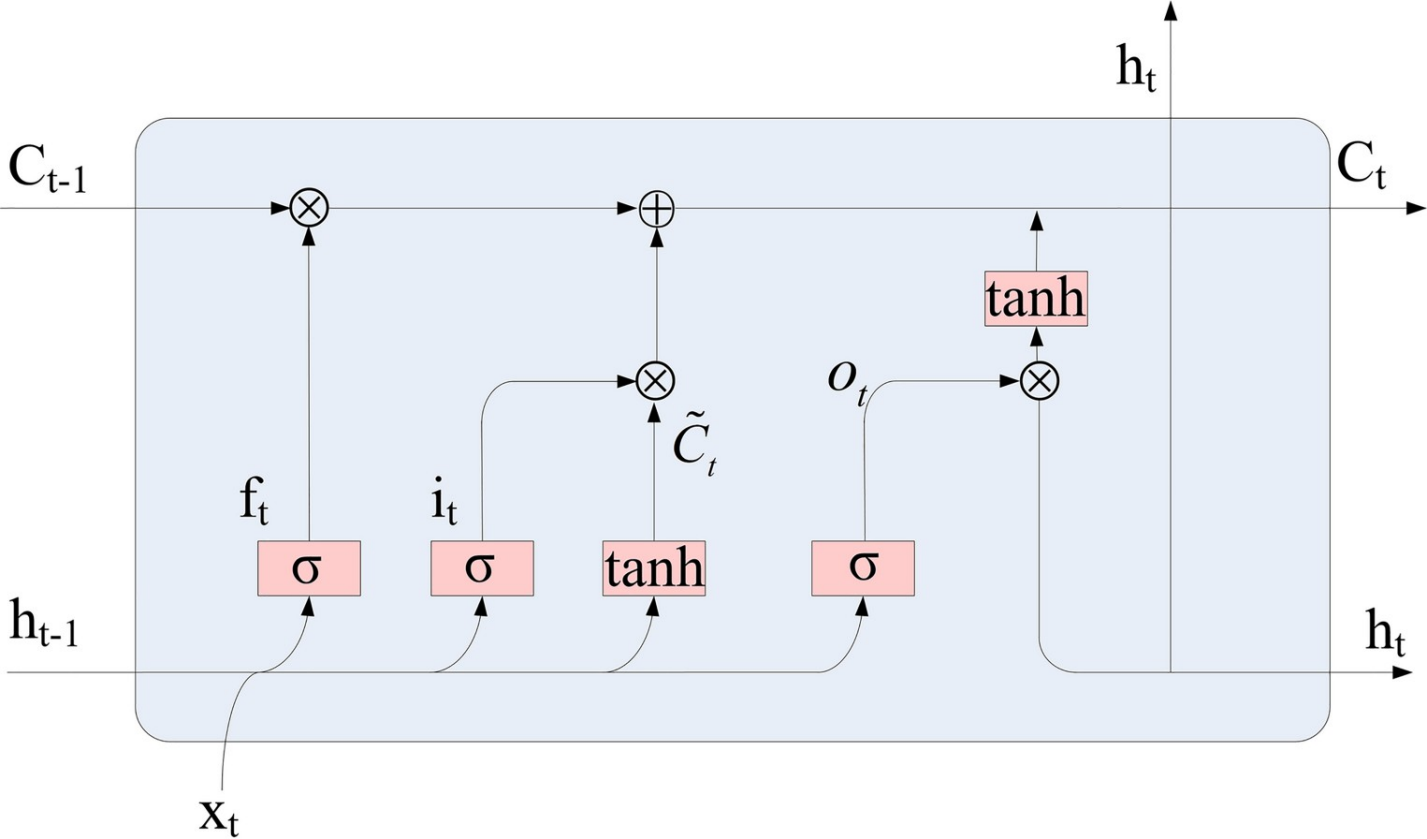
The structure of a LSTM cell.

LSTM was developed from recurrent neural network (RNN) to overcome the gradient vanishing of RNN neural network in long-term time series [51]. LSTM network can delete or add information to the cell state (*c*_*t*_) through a structure called gate (Fig 1). Gates can selectively determine which information can pass through. Compared with RNN network, LSTM can selectively forget part of the historical content after adding the forgetting mechanism [3]. In the forgetting gate (*f*_*t*_), sigmoid units were used to determine what information needs to be discarded in the cell state. The input gate (*i*_*t*_) determines what new information can be added to the cell state, and the output gate (*o*_*t*_) needs to judge what state characteristics of the cell should be output according to the update of the input gate. In addition, compared with the traditional RNN, since LSTM adds a memory unit in the hidden layer to replace the original unit in RNN, feature extraction can be performed on the sequence data with time periods. The mathematical description of the LSTM model update process is as follows [22].


$$C_{t}=f_{t}*C_{t-1}+i_{t}*{\tilde{C}}_{t}$$ (1)

$$h_{t}=o_{t}*tanh(C_{t})$$ (2)

Where *h*_*t*_ refers to hide status, *C*_*t*_ refers to the cell state at time t, *C*_*t*−1_ refers to the cell state at time t-1, ${\tilde{C}}_{t}$ refers to the candidate memory cells, which represents the input information entering the current moment memory cell.

**Fig 2:**
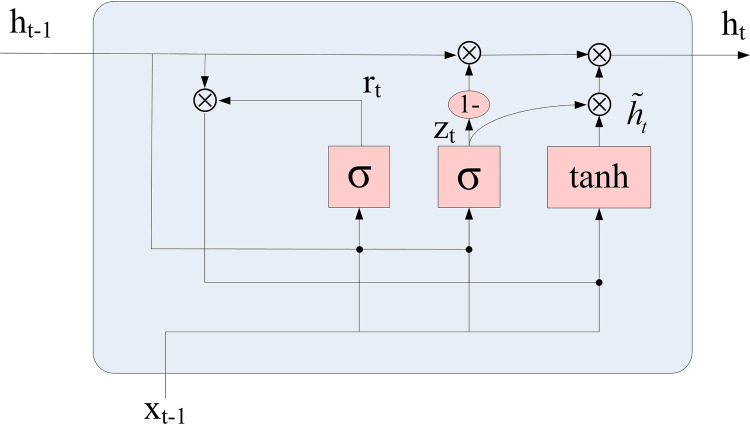
The structure of a GRU cell.

GRU is also a kind of RNN, including update gate (*z*_*t*_) and reset gate (*r*_*t*_) (Fig 2). Therefore, GRU can be seen as a further simplification of the LSTM algorithm. The update gate was used to control what state information in the previous moment (*h*_*t*−1_) can be brought to the current state. The larger the value of the update gate is, the more the state information of the previous time was brought in. The reset gate (*r*_*t*_) was used to control the degree to which the state information of the previous time was ignored. Compared with LSTM, GRU has less gate and less parameters than LSTM, but it can also achieve the same functions as LSTM. The update equations are as shown in Eqs (3)–(5) [52]:

$$r_{t}=\sigma (W_{r}[h_{t-1},x_{t}])$$ (3)

$${\tilde{h}}_{t}=\tanh(W_{\tilde{h}}[r_{t}⊗h_{t-1},x_{t}])$$ (4)

$$h_{t}=(1-z_{t})⊗h_{t-1}+z_{t}⊗{\tilde{h}}_{t}$$ (5)

Where *W*_*r*_ and *W*_*h*_ refer to the network weights matrix, ${\tilde{h}}_{t}$ refers to candidate status

**Fig 3:**
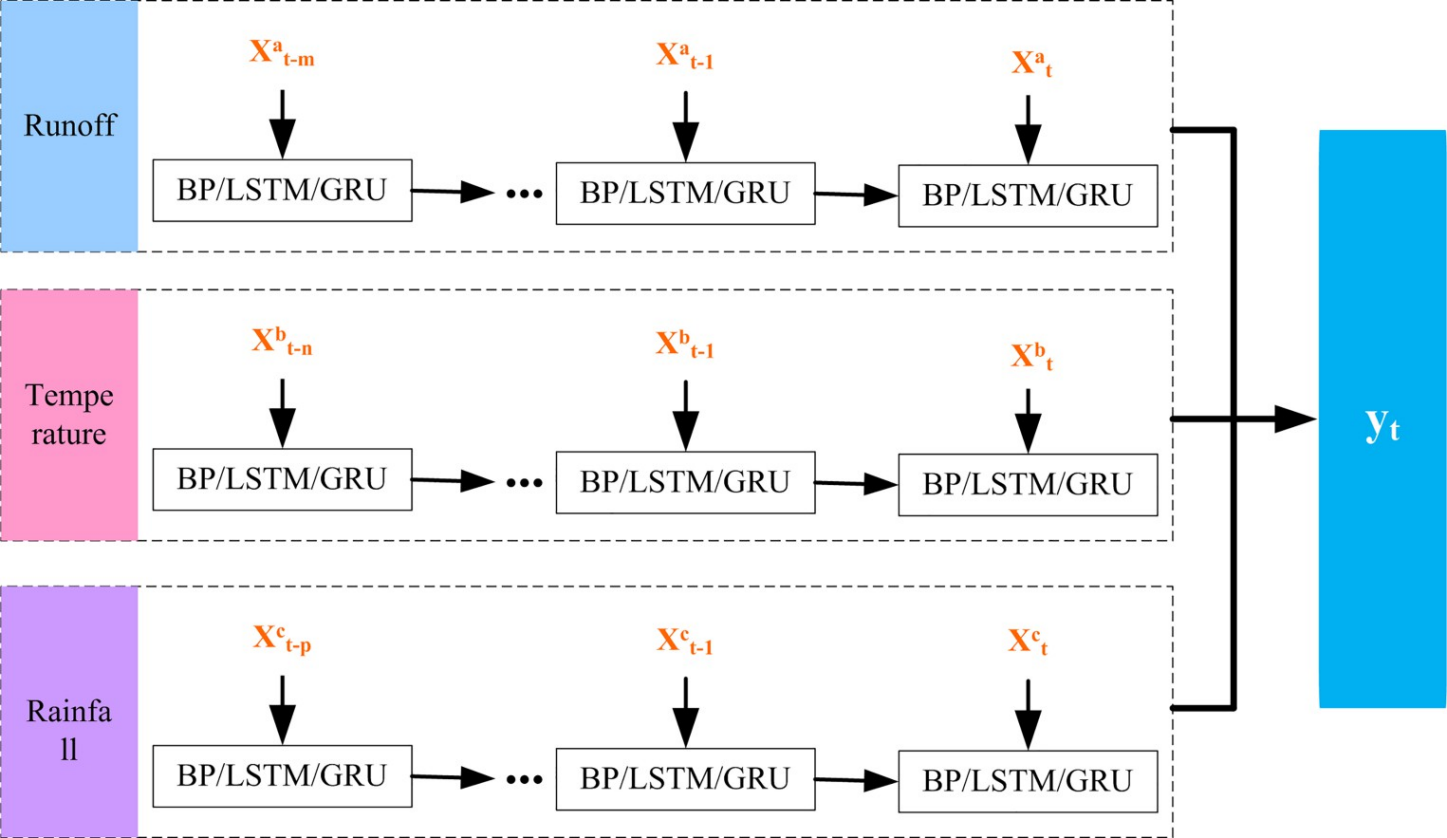
The network architecture of the benchmark model.

As the base sub-model of the TV-DMA module, BP, LSTM and GRU were the foundation to construct the TV-DMA model. Since runoff, rainfall and temperature were used to predict runoff in this study, the network architecture of the benchmark model adopts the network architecture of multiple input and single output. As shown in Fig 3, the input variables include runoff, rainfall and temperature, and the specific input variables were determined by correlation analysis.

In order to ensure the consistency of model comparison, the parameters of the above three machine learning models are calibrated by Monte Carlo sampling. One million groups of parameter combinations were randomly selected within the value range of each parameter to deduce the optimal parameter group of each model. To be specific, the first step is to analyze the value range of all parameters of the k-th model, and randomly generate a set of initial parameter combinations [*σ*_1_, *σ*_2_, …,*σ*_*m*_]. Secondly, take Root Mean Square Error (RMSE) as the objective function, and calculate the RMSE of the simulation results under this parameter combination. Repeat the above steps for 1million times to randomly generate 1million groups of parameter combinations and calculate the RMSE under each parameter combination. On this basis, the minimum parameter combination of RMSE was selected as the parameter of the model. Finally, repeat the above steps for all models to calibrate their parameters.

### TV-DMA model

**Fig 4:**
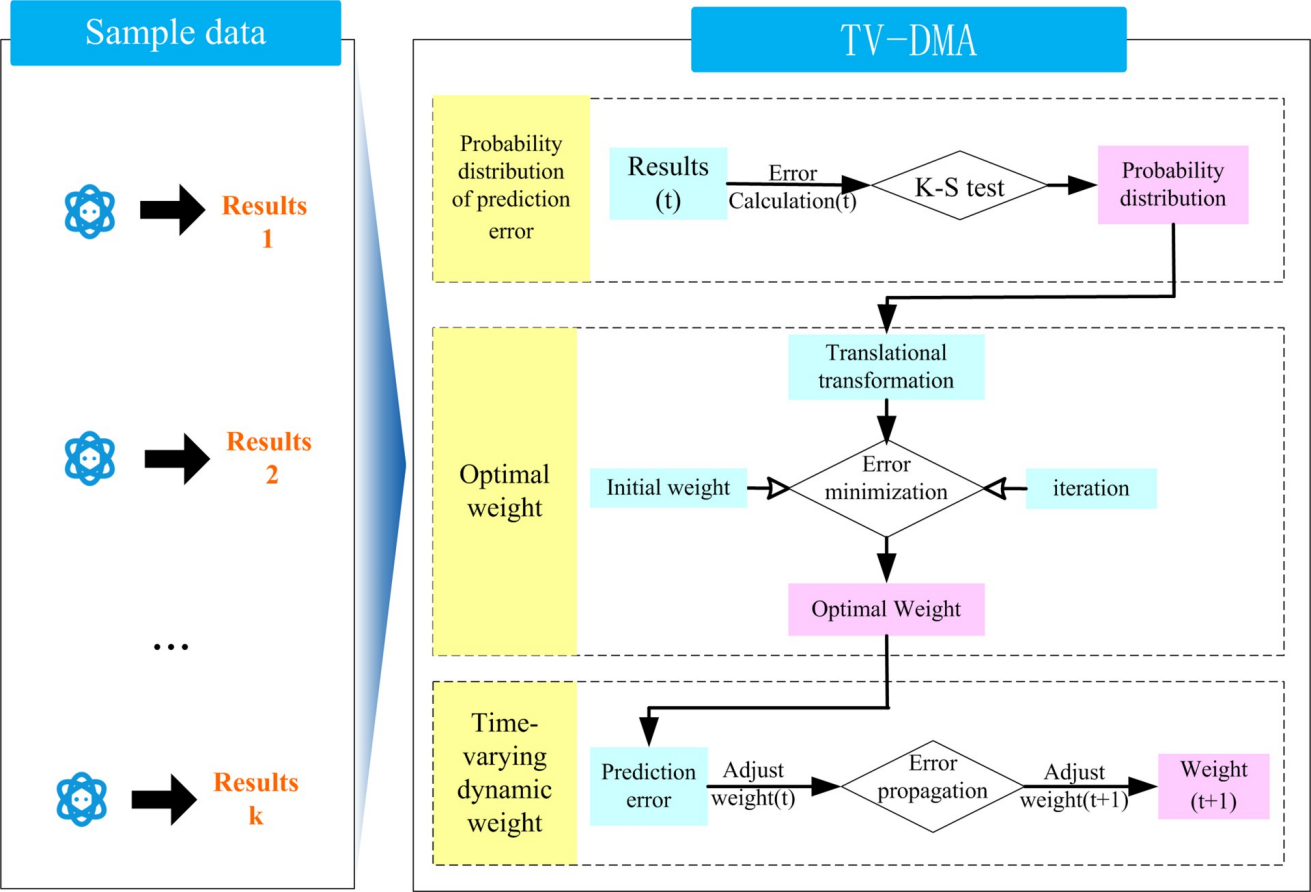
The structure of TV-DMA.

In this section, the TV-DMA model was proposed to improve the ability of the deep learning model in runoff prediction. The key of model averaging method is to determine the weight of single model. In this study, the time-varying weight was derived by translation transformation of the probability distribution, minimizing the local error criterion and the error propagation mechanism. As shown in Fig 4, the TV-DMA method mainly includes three key steps. Step 1 is to use the error distribution characteristics of the prediction results and Kolmogorov-Smirnov test (K-S) to calculate the probability distribution of the prediction results of each model. Step 2 is to calculate the weight of each model by using the translation transformation of the distribution function and the criterion of minimizing local error. Step 3 is to use the error propagation mechanism to dynamically adjust the weight of the current time, and synthesize the time-varying weights of each model to obtain the integrated prediction results. The detailed implementation process and method description were as follows:

**Step1**: Probability distribution of the prediction results

Assuming that *y* is the predicted runoff, *D* = [*y*_1_,*y*_2_,……,*y*_*T*_] is the measured runoff data required for calibration model, and *e* represents the error between the prediction results and the measured results. And the K-S test was used to identify the distribution characteristics of the prediction error of each model.


$$e=[e_{1},e_{2},\ldots \ldots ,e_{k}]$$ (6)

$$e_{k}=[e_{k}^{1},e_{k}^{2},\ldots ,e_{k}^{T'}]$$ (7)

Where *e*_*k*_ refers to the prediction error of the *k*-th model, $e_{{}_{k}}^{T'}$ refers to the prediction error of the *k*-th model in the *T*-th category. As the runoff forecast targeted in this study, $e_{{}_{k}}^{T'}$ refers to the prediction error of the *k*-th model in each month.

**Step2**: Optimal weight of single model

According to the identified model error, the distribution function translation transformation was used to correct the prediction results of single model. Assuming that the error $e_{{}_{k}}^{T'}$ of the *k*-th model in *T*-th category obeys the normal distribution $N∼(\mu_{k}^{T'},\sigma_{k}^{T'})$, then the error $e_{{}_{k}}^{T'}$ obeys new normal distribution $N∼(0,\sigma_{k}^{T'})$ after the distribution function translation transformation obeys the normal distribution. On this basis, the sum of squares of errors (*SSE*) was used to determine the initial weight value $\omega_{k}^{\left(0\right)}$ of each model.


$$\omega_{k}^{\left(0\right)}=\frac{{\left(SSE_{k}^{T'}\right)}^{-1}}{\sum_{k=1}^{3}{\left(SSE_{k}^{T'}\right)}^{-1}}$$ (8)

According to the initial weight value, the initial result ${\hat{y}}^{\left(0\right)}$ of the integrated prediction and the error $\left({\hat{y}}^{\left(0\right)}-y\right)$ of the integrated prediction were calculated by weighted average. As there is some error in the integrated prediction results of initial weights, the optimal weights were determined by using the minimization error criterion and iterative method to reduce the error as much as possible. Specifically, the weights $\omega_{k}^{\left(1\right)}$ of each model were adjusted according to the initial error of the integrated prediction results, and the iterative method was used to repeat the process of Eq 10. The weight $\omega_{k}^{\left(n\right)}$ of the benchmark model is finally obtained with the goal of minimizing the local error.

$${\hat{y}}^{\left(0\right)}=\sum_{k=1}^{3}\omega_{k}^{\left(0\right)}(y_{k}^{T'}-\mu_{k}^{T'})$$ (9)

$$\omega_{k}^{\left(1\right)}=\{\begin{array}{l} \omega_{\min(y_{1},y_{2},\ldots ,y_{k})}^{\left(0\right)}-\frac{{\left({\hat{y}}^{\left(0\right)}-y\right)}^{2}}{\sum_{k=1}^{3}{\left(SSE_{k}^{T'}\right)}^{-1}}\;({\hat{y}}^{\left(0\right)}-y)>0\\ \omega_{\max(y_{1},y_{2},\ldots ,y_{k})}^{\left(0\right)}+\frac{{\left({\hat{y}}^{\left(0\right)}-y\right)}^{2}}{\sum_{k=1}^{3}{\left(SSE_{k}^{T'}\right)}^{-1}}\;({\hat{y}}^{\left(0\right)}-y)<0 \end{array}$$ (10)

$$\omega_{k}^{\left(n\right)}=[\omega_{1}^{\left(n\right)},\omega_{2}^{\left(n\right)},\ldots ,\omega_{k}^{\left(n\right)}]\;k=3$$ (11)

**Step3**: Dynamic adjustment of weight

Although the optimal weights of each model were obtained by using the translational transformation of distribution function and the minimum error criterion, the static weights were difficult to adapt to the changes of time series because of the large uncertainty of runoff series. In addition, the prediction of time series for the next time depends on the results and performance of the current time, which has obvious dependence characteristics. Therefore, in order to deal with this phenomenon, the error propagation mechanism was introduced to dynamically adjust the weight of the model, that is, the weight of the next time was adjusted by using the prediction error change range of the model average method at the previous time in equal proportion.

Assuming that the optimal weight at the current time was determined to be $\omega_{k}^{\left(n\right)}$, the prediction result at the current time was ${\hat{y}}_{t}^{\left(n\right)}$. Due to the influence of input uncertainty, new errors *ε* were generated when using this weight for prediction. Therefore, the iterative method (Eq 12) was used to determine the new weights ${\tilde{\omega }}_{k}$ and the change range *λ*_*k*_ of the weight of each model. On this basis, the weight at the next time was adjusted in equal proportion to obtain the prediction results at the next time. Finally, repeat this step at each time prediction until the prediction task ends.


$${\hat{y}}_{t}^{\left(n\right)}=\overset{k=1}{\underset{3}{\sum }}\omega_{k}^{\left(n\right)}(y_{k}^{T'}-\mu_{k}^{T'})$$ (12)

$$\lambda_{k}=\frac{\left({\tilde{\omega }}_{k}-\omega_{k}^{\left(n\right)}\right)}{\omega_{k}^{\left(n\right)}}$$ (13)

$${\hat{y}}_{t+1}^{\left(n\right)}=\lambda_{k}\overset{k=1}{\underset{3}{\sum }}\omega_{k}^{\left(n\right)}(y_{k}^{T'}-\mu_{k}^{T'})$$ (14)

### Uncertainty analysis of the model

In this study, Monte Carlo sampling method was used to derive the uncertainty interval of TV-DMA model prediction results. Firstly, a randomly generated integer from 1 to k was used to sample model, then the prediction value of runoff was randomly generated according to the probability distribution of the selected model at time t. Repeat the above steps for 10000 times to obtain 10000 samples at any time t. Rank these samples, and the 95% prediction interval of the TV-DMA model is the part between 2.5% and 97.5% quantiles. Similarly, the above method was also used to calculate the uncertainty of the single model. Select the interval band (B) and coverage rate (CR) to evaluate the uncertainty of the model. The higher the CR is, the narrower the B is, indicating that the model uncertainty is smaller. On the contrary, the model uncertainty is larger.

### The performance evaluation method

In order to evaluate the performance of the model in runoff prediction, the mean average absolute (MAE) and Nash-efficiency coefficient (NSE) were selected to evaluate the accuracy of the model. MAE reflects the deviation degree of runoff prediction, and NSE reflects the coincidence degree between the predicted runoff process and the measured runoff process.


$$MAE=\frac{\sum_{i=1}^{n}(x_{i}-y_{i})}{n}$$ (15)

$$NSE=1‐\frac{\sum_{i=1}^{n}(x_{i}-y_{i}{)}^{2}}{\sum_{i=1}^{n}(y_{i}-\bar{y}{)}^{2}}$$ (16)

Where *x*_*i*_ is the predicted runoff, *y*_*i*_ is the measured runoff, and $\bar{y}$ is the mean value of the measured runoff.

## Results

### Catastrophe analysis of runoff series

**Fig 5:**
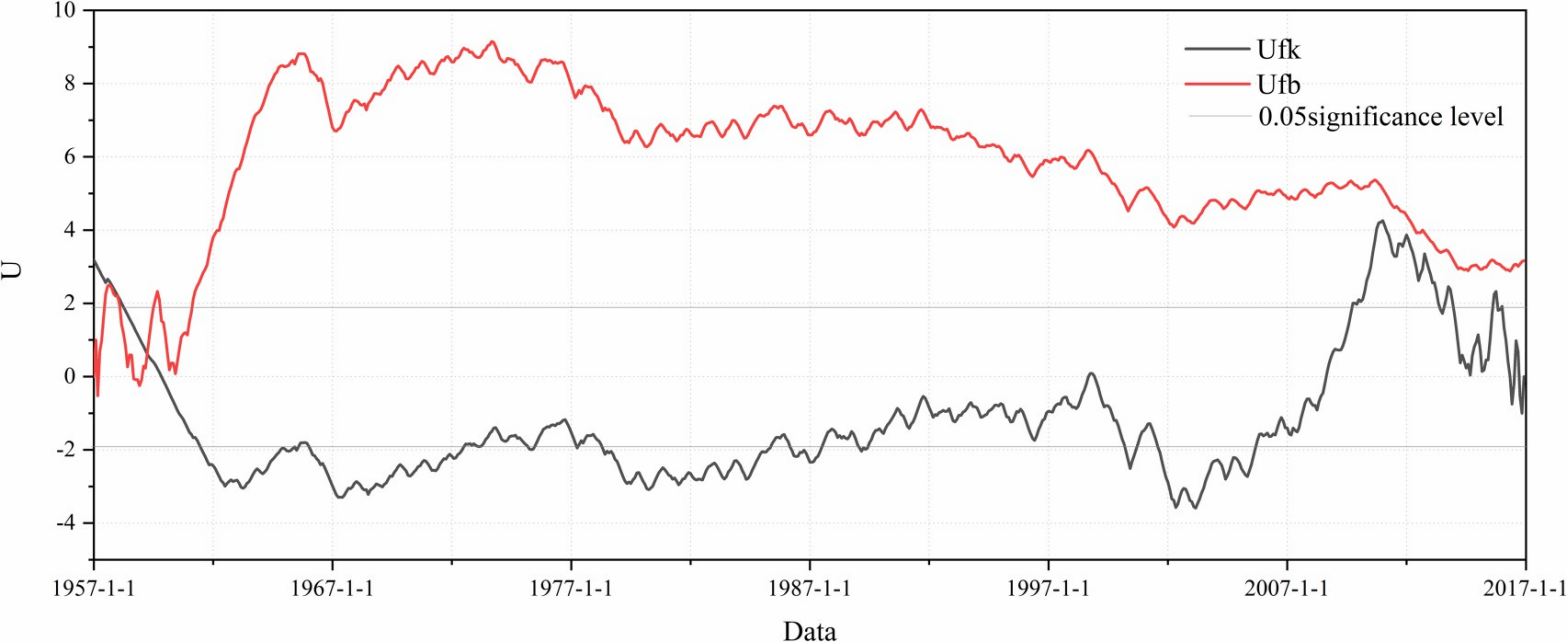
The abrupt characteristics of runoff series.

The abrupt change of runoff sequence will cause more uncertainty to runoff prediction. Therefore, this study uses M-K test method to analyze the abrupt characteristics of runoff series, which aims to determine the sample data of runoff prediction. M-K test is a classical mutation point test method. By drawing the statistical sequence curve, M-K test can analyze whether there is a mutation point and the occurrence time of the mutation point at 0.05 significance level. As shown in Fig 5, the abrupt change of runoff series occurred around 1959, and the runoff has shown a downward trend since 1974 (from the Ufk curve), when it was the time to carry out industrialization reform and Reform and Open policy to promotes the construction of water conservancy projects [4]. Therefore, this study selects the runoff series data after 1960 as the sample data of the model.

### Variables selection of runoff prediction

**Fig 6:**
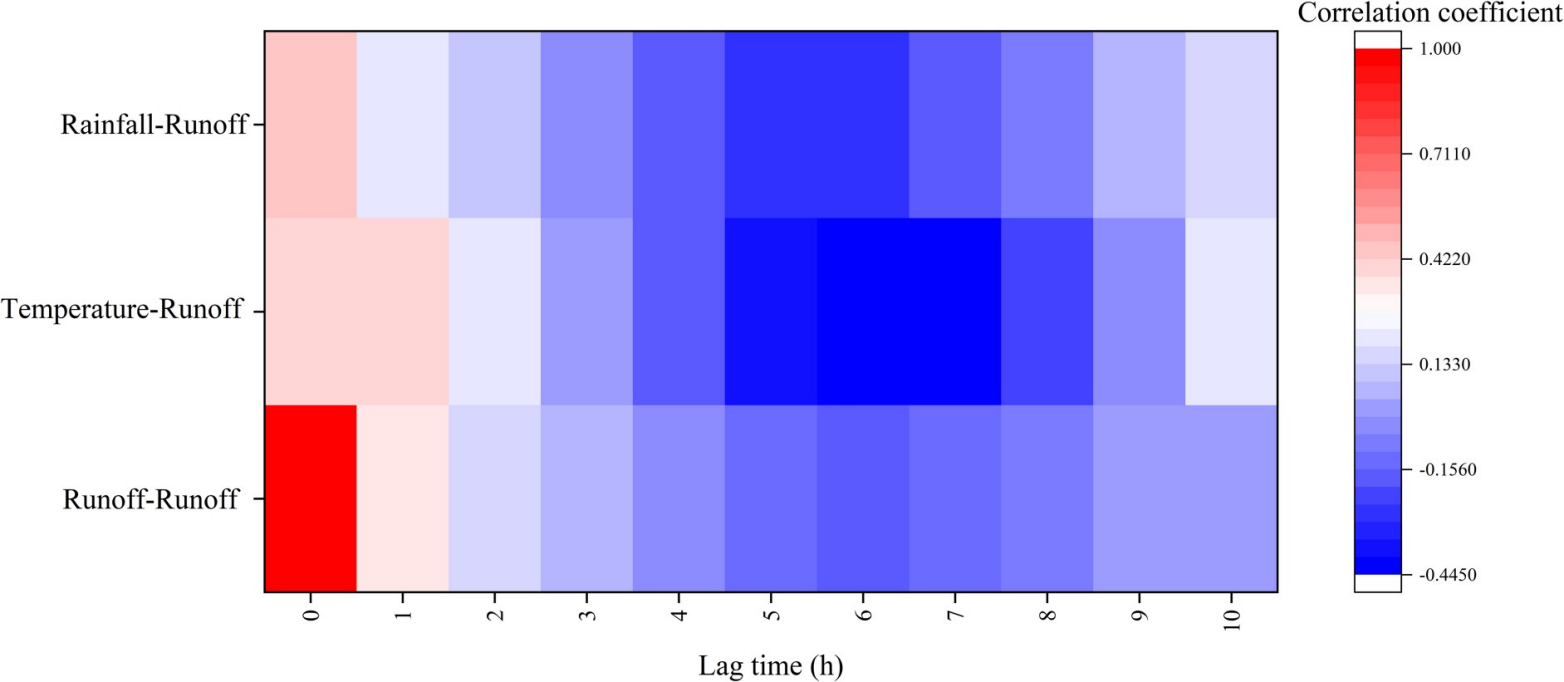
Correlation of runoff prediction variables.

The selection of runoff prediction variables is one of the key steps of runoff prediction. Index combination method and correlation analysis method were often used to select input variables [53]. Index combination usually tests several combinations of input variables, including some available meteorological variables [54]. Although this method is a search strategy with high computational efficiency, only checking a part of all combinations will still make people doubt whether there are some combinations with low data requirements that are better than the selected best combination [53]. However, the input variable selection method based on correlation analysis can statistically quantify the correlation between each variable and runoff, and screen the input variables according to the maximum correlation, which is often used in the selection of input variables in runoff prediction [55]. Correlation analysis methods mainly include correlation coefficient, principal component analysis (PCA), chart analysis, significance test, regression analysis and information entropy. Among them, the correlation coefficient method can quantitatively give the correlation between variables, which is often used in the selection of input variables. Therefore, the correlation coefficient approach was used to select the input variables in this study. Considering the hysteresis and correlation of variable factors, this study selects variables with correlation coefficient greater than 0.3 as runoff prediction variables. As shown in Fig 6, the variables with correlation coefficient greater than 0.3 were: current monthly runoff, runoff of the previous month, current monthly temperature, temperature of the previous month and current monthly rainfall. Therefore, these variables were selected the driving factors of runoff prediction.

### Runoff prediction results of BP, LSTM and GRU models

**Fig 7:**
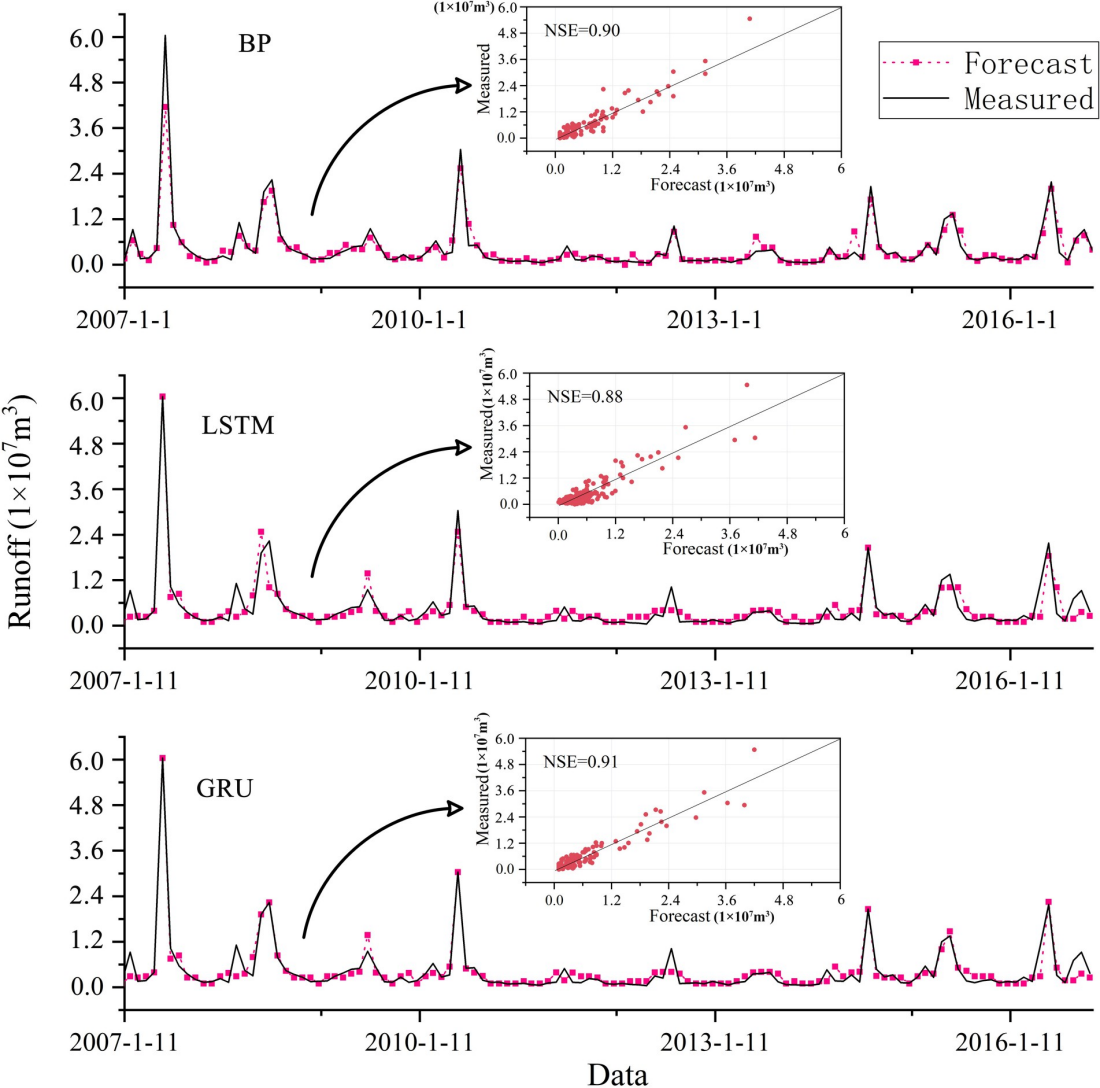
Runoff prediction results of BP, LSTM, and GRU model.

**Fig 8:**
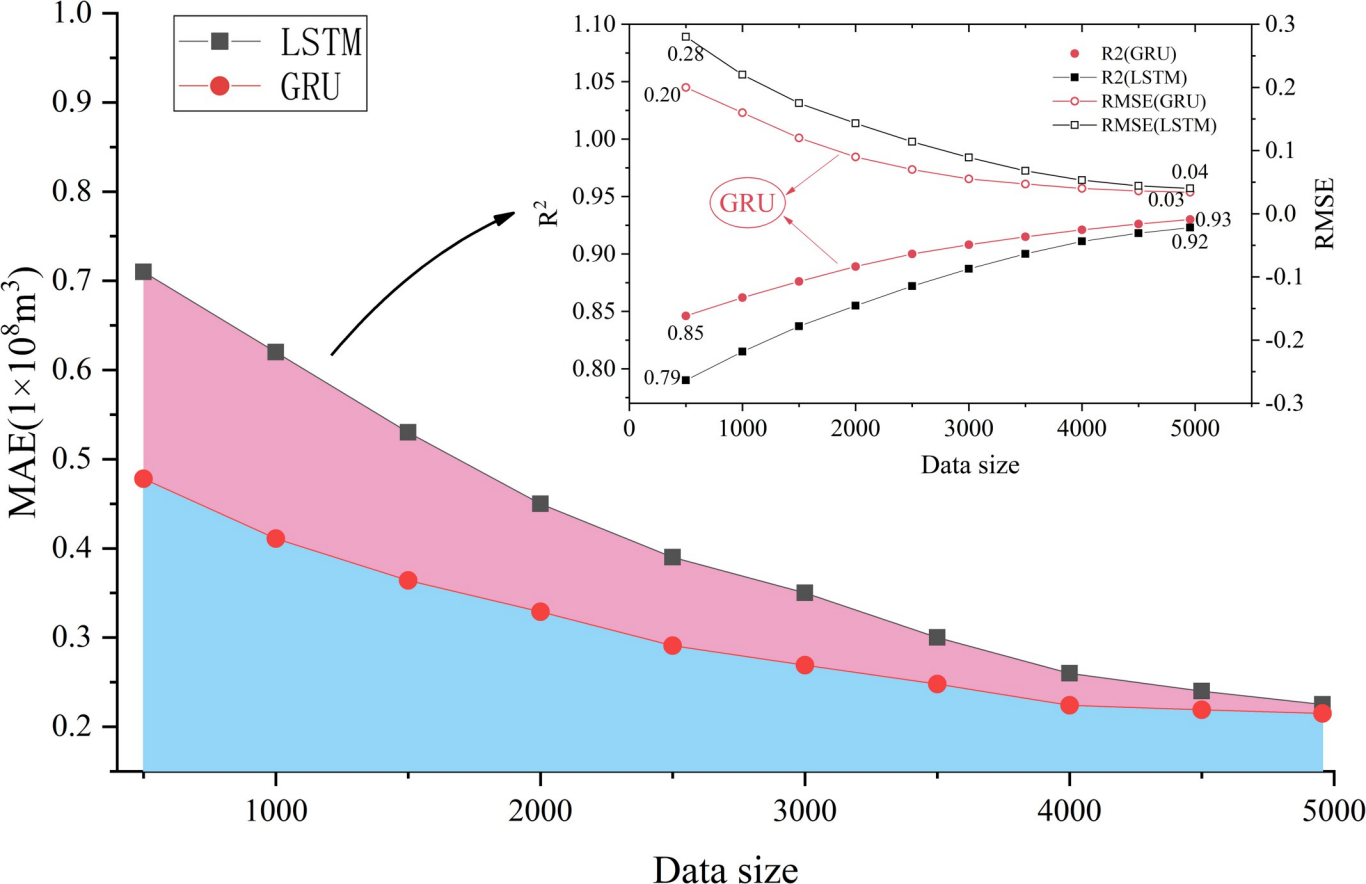
The sensitivity between LSTM and GRU model to data size.

The monthly runoff data from 1961 to 2016 in Xixian hydrologic station were used to construct the monthly runoff prediction model. The runoff data from 1961 to 2006 were used as training data, and the runoff data from 2007 to 2016 were used to verify the performance and generalization ability of the model. As shown in Fig 7, the NSE of these single models (BP, LSTM, and GRU) are above 0.88, which demonstrate that these single models show high accuracy in runoff prediction. Among them, the NSE of the prediction result of GRU model was 0.91, while the NSE of the prediction result of LSTM model was 0.88, which indicates that the performance of GRU model is better than that of LSTM model under the current sample size. In order to deeply analyze the sensitivity of GRU and LSTM to data size in runoff prediction. The difference of prediction performance between GRU and LSTM was analyzed by changing the sample size. As shown in Fig 8, the root mean square error (RMSE) of LSTM model was 40% higher than that of GRU model under small sample size, and the Correlation coefficient is 0.06 lower than that of GRU model, which shows that the prediction performance of LSTM model is obviously lower than GRU model in small sample size. However, with the increase of sample size, the prediction performance of LSTM model shows a steady upward trend. When reaching the maximum sample size, the difference in the Correlation coefficient between LSTM model and GRU model is only 0.01, which demonstrate that the predictive performance of the LSTM model and the GRU model is almost no difference under current sample size. The main reason is that GRU has fewer parameters than LSTM, which makes it easier to converge in fewer samples [3]. These results demonstrate that the prediction performance of LSTM model is more sensitive to the sample size in the runoff prediction, and the prediction performance increases with the increase of the sample size. However, the prediction performance of GRU model is better than that of LSTM model under different data size.

In addition, it can be easily found that these single models have certain differences in prediction at different times. For example, the LSTM model obviously underestimated the runoff in the flood season in 2007. BP model has a high prediction effect in flood season in 2007, but it has a poor prediction effect in flood season in 2008. The prediction effect of GRU model in non-flood seasons shows higher accuracy, but the prediction effect of runoff in flood seasons in 2008, 2012 and 2016 is significantly reduced. Therefore, it can be found that there is always some uncertainty in the runoff prediction by single model. It is necessary to use the model average method to build an ensemble forecasting model to improve the stability of the runoff prediction [56].

### Runoff prediction results of TV-DMA model

**Fig 9:**
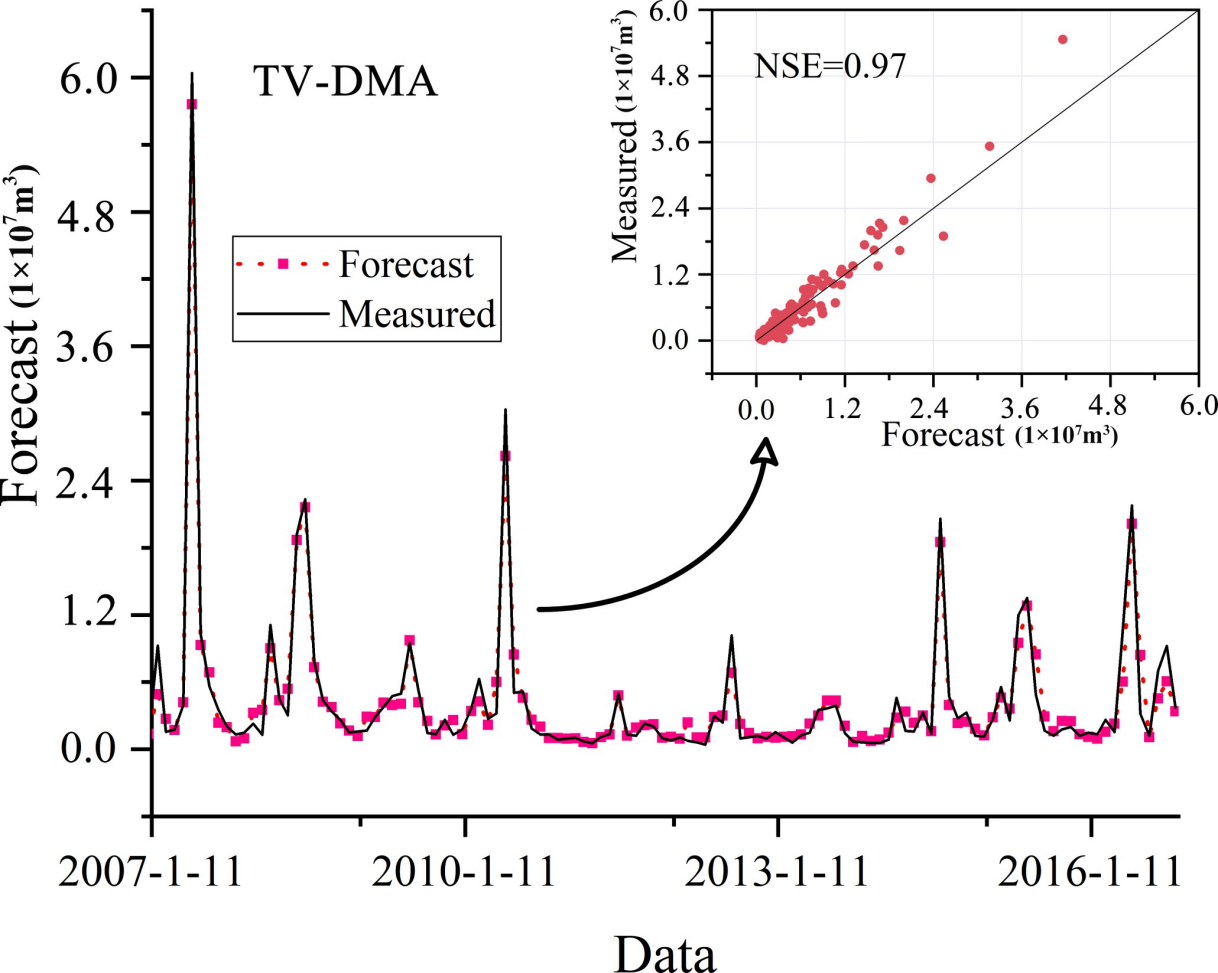
Runoff prediction results of TV-DMA model.

In order to reduce the prediction uncertainty of single model, an integrated prediction model for runoff prediction was constructed by TV-DMA algorithm, which weighted average the prediction results of BP, LSTM and GRU model. As shown in Fig 7, the predicted results of TV-DMA model fit the actual runoff well, and the NSE of TV-DMA model reaches 0.97, which is significantly higher than the single model. In addition, the scatter diagram in Fig 9 demonstrates that the TV-DMA model has good prediction effect for small runoff, but there is some uncertainty in the prediction of large runoff. The main reason is that the runoff and the fluctuation of runoff series in non-flood seasons is small. The TV-DMA model can accurately capture the characteristics of runoff series. However, the runoff and the fluctuation of runoff series in flood seasons is large, which makes the model have some deviation. Nevertheless, the results in Figs 7 and 9 indicate that the prediction performance of TV-DMA is significantly better than that of single model, which can provide higher accuracy and more stable runoff prediction.

**Fig 10:**
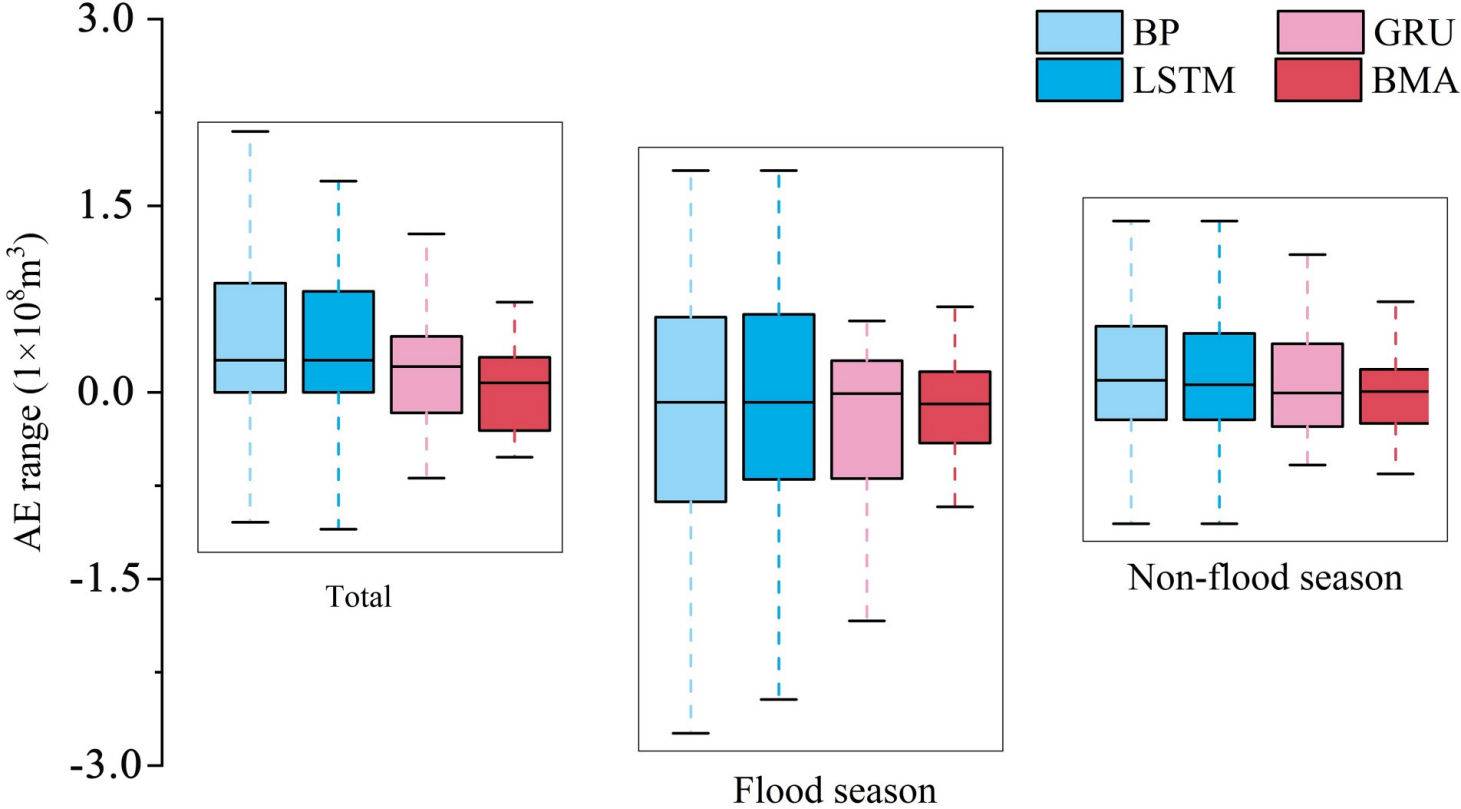
Accuracy difference of prediction in different periods among single model and TV-DMA model.

In order to further analyze and compare the monthly runoff prediction characteristics of each model, the accuracy difference between single model and TV-DMA model in flood season, non-flood season and the whole process was analyzed. As shown in Fig 10, whether it is single model or TV-DMA model, the prediction error in flood season is significantly higher than that in non-flood season. From the characteristics of runoff series, the runoff in flood season fluctuates greatly, and it is difficult for data-driven models to accurately capture the nonlinear relationship of such violent fluctuations under limited samples. In addition, the intensity of human regulation of runoff in flood season is significantly increased, which increases the uncertainty of runoff prediction in flood season. In terms of model accuracy, GRU model has low prediction error among the three single models. However, TV-DMA model always has the lowest error in flood season, non-flood season and the whole continuous process, which demonstrates that TV-DMA model effectively integrates the characteristics of single model prediction and has more stable prediction ability in runoff prediction.

## Discussion

### Model uncertainty analysis

As shown in Table 1, whether it is single model or TV-DMA model, the interval band of runoff forecast in flood season is significantly higher than that in non-flood season, and the CR in flood season is lower than that in non-flood season, which indicates that the proposed method has higher uncertainty in flood season prediction. The main reason is that the reservoir needs to realize the engineering purpose of power generation and flood control in flood season, its regulation and storage range was significantly higher than that in non-flood season, affecting hydrological conditions, river ecology and natural flow [57]. In addition, compared with three single models, TV-DMA model always has the narrowest interval band, which is 33.3%-65.5% lower than three single models. Therefore, although TV-DMA has certain uncertainty in runoff prediction, TV-DMA model significantly reduces the uncertainty of the prediction results of the single model and can provide more stable runoff prediction [58].

**Table 1:** Uncertainty analysis results of different models.

Method	Flood season		Non-Flood season		Total	
	CR (%)	B (1×10^8^m^3^)	CR (%)	B (1×10^8^m^3^)	CR (%)	B (1×10^8^m^3^)
BP	95.1	0.03	96.3	0.027	95.7	0.029
LSTM	95.2	0.021	96.9	0.021	96.1	0.021
GRU	95.7	0.016	97.4	0.013	96.6	0.015
TV-DMA	96.3	0.011	98.6	0.009	97.5	0.010

### Feature importance analysis

**Fig 11:**
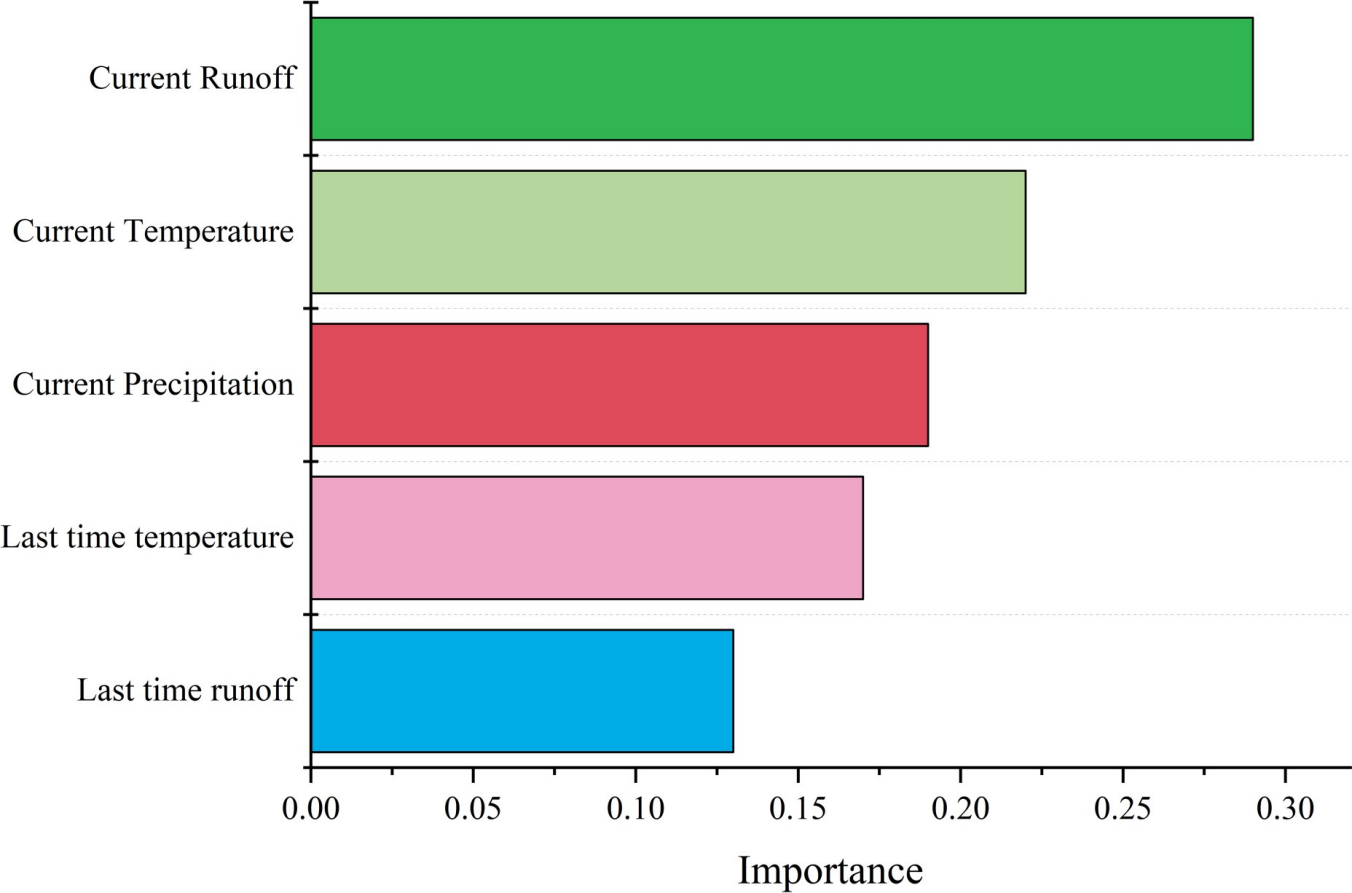
Feature importance analysis of the driving factors.

The driving factor in runoff prediction is an important factor affecting runoff prediction. In order to analyze the influence of different driving factors on runoff prediction, the contribution of current monthly runoff, runoff of the previous month, current monthly temperature, temperature of the previous month and current monthly rainfall to runoff prediction was calculated. As shown in Fig 11, the current monthly runoff is the most important factor affecting the runoff prediction. The main reason is that the runoff prediction is a time series prediction problem. Time series prediction has a strong dependence on the state of the previous time. Therefore, the current monthly runoff is the most important driving factor in runoff prediction. In addition, it can be found that the current monthly temperature and rainfall also have a great impact on runoff prediction. Because rainfall and temperature will change the catchment characteristics and flow in the basin, which will affect the runoff prediction. On the contrary, runoff and temperature of the previous month have little effect on runoff prediction. The reason may be that the runoff and temperature at the previous moment will affect the runoff at the current time, but have small correlation with the runoff at the next moment. Therefore, it can be concluded that in the runoff forecast and control, flood control managers should not only pay attention to the current runoff, but also pay attention to the influence of rainfall and temperature.

## Conclusions

Based on the proposed TV-DMA approach, this study constructed an integrated prediction framework for runoff prediction. On the basis of analyzing the characteristics of runoff series, this study proposed the main variables suitable for runoff prediction, and constructed an integrated runoff prediction model using TV-DMA model and three neural network models. The main conclusions are as follows:

(1) The runoff sequence of Xixian station in Huaihe River Basin changed suddenly around 1959, and the runoff has shown a downward trend since 1974.

(2) The current monthly runoff, the runoff of the previous month, the current monthly temperature, the temperature of the previous month and the current monthly rainfall were the main driving factors suitable for runoff prediction.

(3) TV-DMA model effectively integrates the characteristics of single model to provide higher accuracy prediction. The NSE of the prediction result of TV-DMA model reaches 0.97, which is 6.6%-10.2% higher than that of single model.

(4) The results of model uncertainty analysis demonstrate that the interval band of TV-DMA model was 33.3%-65.5% lower than that of single model, which indicate that TV-DMA significantly reduced the uncertainty of the prediction results.

(5) The current monthly runoff, rainfall and temperature are the important factor affecting the runoff forecast, which should be paid special attention in the runoff prediction.

However, due to the limitation of sample data, this study only considers the impact of rainfall, runoff and temperature on runoff prediction. Future study can explore the factors affecting runoff prediction to improve the performance of runoff prediction to improve the performance of runoff prediction. Nevertheless, this study proposed a dynamic model averaging method with Time-varying coefficients, which has great potential in mobility. On the one hand, this approach can be used not only for coupling machine learning models, but also for coupling numerical models based on physical mechanisms. On the other hand, this approach can not only be used to provide more robust runoff prediction, but also be used in urban flood prediction, rainfall prediction and other prediction tasks.

## Supporting information

S1 Data (XLSX)
