# Correction: Improved runoff forecasting based on time-varying model averaging method and deep learning

**DOI:** 10.1371/journal.pone.0307923

**Published:** 2024-07-24

**Authors:** Jinlou Ruan, Yang Cui, Kai Xiang, Yuchen Song

The first author’s name is spelled incorrectly. The correct name is: Jinlou Ruan. The correct citation is: Ruan J, Cui Y, Xiang K, Song Y (2022) Improved runoff forecasting based on time-varying model averaging method and deep learning. PLOS ONE 17(9): e0274004. https://doi.org/10.1371/journal.pone.0274004
